# Synthesis of Si/Fe_2_O_3_-Anchored rGO Frameworks as High-Performance Anodes for Li-Ion Batteries

**DOI:** 10.3390/ijms222011041

**Published:** 2021-10-13

**Authors:** Yajing Yan, Yanxu Chen, Yongyan Li, Xiaoyu Wu, Chao Jin, Zhifeng Wang

**Affiliations:** 1 School of Materials Science and Engineering, Hebei University of Technology, Tianjin 300401, China; 201921801024@stu.hebut.edu.cn (Y.Y.); yxchen116@163.com (Y.C.); xywuhebut@163.com (X.W.); jinchaohebutmail@163.com (C.J.); 2 Key Laboratory for New Type of Functional Materials in Hebei Province, Hebei University of Technology, Tianjin 300401, China; 3 Research Institute of Foundry, Hebei University of Technology, Tianjin 300401, China

**Keywords:** dealloying, Si, Li-ion battery, anode, nanoporous

## Abstract

By virtue of the high theoretical capacity of Si, Si-related materials have been developed as promising anode candidates for high-energy-density batteries. During repeated charge/discharge cycling, however, severe volumetric variation induces the pulverization and peeling of active components, causing rapid capacity decay and even development stagnation in high-capacity batteries. In this study, the Si/Fe_2_O_3_-anchored rGO framework was prepared by introducing ball milling into a melt spinning and dealloying process. As the Li-ion battery (LIB) anode, it presents a high reversible capacity of 1744.5 mAh g^−1^ at 200 mA g^−1^ after 200 cycles and 889.4 mAh g^−1^ at 5 A g^−1^ after 500 cycles. The outstanding electrochemical performance is due to the three-dimensional cross-linked porous framework with a high specific surface area, which is helpful to the transmission of ions and electrons. Moreover, with the cooperation of rGO, the volume expansion of Si is effectively alleviated, thus improving cycling stability. The work provides insights for the design and preparation of Si-based materials for high-performance LIB applications.

## 1. Introduction

Over the past few decades many energy storage devices have been designed to meet the needs of electric vehicles and portable electronic devices in the context of rapid fossil fuel consumption [1,2,3,4,5,6]. Lithium-ion batteries (LIBs) are receiving increasing attention on account of their long life, high energy density, and environmental friendliness [7,8,9,10,11,12,13]. However, it is urgent to explore novel electrode materials to meet the demand for higher-energy-density LIBs. Among the various candidates, Si is an exciting and promising anode candidate for the development of high-performance LIBs because of its high theoretical mass specific capacity (4200 mAh g^−1^) and low operating voltage [14,15,16]. Unfortunately, large volume variations, structural fragmentation, and the cracking of Si anodes during repeated charge/discharge processes result in severe capacity attenuation and electrical contact loss, impeding their widespread commercial application [17,18].

Some strategies have been suggested to enhance the overall property of Si anodes. One effective strategy is to synthesize Si anodes with different nanostructures, such as Si nanowires, nanotubes, nanoparticles, and so on [19,20,21]. Mueller et al. [22] prepared Si nanoparticle-loaded graphite micron particles by a fluidized bed granulation method, showing high Li storage properties. Zhang et al. [23] synthesized P-doped porous Si nanoparticles by magnesiothermic reduction, resulting in 1761 mAh g^−1^ at 0.5 A g^−1^ after 80 cycles. The above Si nanoparticles with the advantages of small size and high specific surface area can provide a transfer path and sufficient room for the rapid transmission of Li^+^, which is beneficial to reduce the cracking and grinding of electrodes. Another effective strategy is to synthesize different Si/C composite materials [24,25,26]. Graphene and reduced graphene oxide (rGO) with high electrical conductivity and high mechanical strength have been adopted in the design of Si-C anodes for LIBs to relieve the large stress caused by continuous charge–discharge cycles [27,28]. For example, vertically aligned Si@rGO frameworks were prepared by Park et al. [29] through a series of processes including gelation, freeze-casting, magnesiothermic reduction, acid etching, and thermal carbon coating. Capacity retention of 68% could be obtained after 150 cycles. Si nanoparticle-encapsulated GO nanoribbons were prepared by Yao et al. [30] through an electrostatic-induced self-assembly process, presenting values of 1235 mAh g^−1^ at 1 C after 500 cycles. Furthermore, an additional potential strategy is the combination of Si with an anode material containing relatively low capacity, which may relieve the internal stress originating from the severe volume variation of anodes during cycling. Im et al. [31] prepared polylaminate 2D nanoparticles with alternately arranged Si and SiO_x_ layers, displaying a capacity retention of 88% after 200 cycles. Liu et al. [32] synthesized Si@Fe_3_O_4_@FLG as anode materials for LIBs, demonstrating an excellent capacity of 637 mAh g^−1^ at 2 A g^−1^ after 1600 cycles. However, most of the previously revealed preparation methods are complex, costly, result in low yield, and show difficulties in mass production. As a result, it is highly necessary to exploit a simple and low-cost route to fabricate Si-based anodes.

In our previous study, low-cost Al ingots and natural ferrosilicon ores were selected as the starting raw materials to prepare dual-network porous Si/Al_9_FeSi_3_/Fe_2_O_3_ composites [33]. The fabrication process combined melt-spinning and free dealloying. This method involves low-cost initial materials, a simple process, easy scaling, and promise for mass production [34]. In this process, Al_9_FeSi_3_ with a relatively low specific capacity (839.7 mAh g^−1^) was obtained. This was clearly lower than that of Si and Fe_2_O_3_ (1007 mAh g^−1^) and limited the development of high-performance anodes. In our improved case, ball milling was utilized for melt-spinning ribbons prior to dealloying treatment [35]. As a result, a porous Si/Fe_2_O_3_ dual network anode (free of Al_9_FeSi_3_) was prepared by changing the elemental distribution and phase composition of the dealloying precursors. Although the composition of the dealloyed product was optimized, its electrical conductivity and electrochemical performance need to be further improved. To settle this concern, an rGO network was introduced into the Si/Fe_2_O_3_ anode by two kinds of methods in this study. It was found that the as-synthesized Si/Fe_2_O_3_ anchored rGO framework by the ball milling route presented significantly improved Li storage properties and cycling stability as the anode for LIBs, delivering 1744.5 mAh g^−1^ at 200 mA g^−1^ after 200 cycles and 889.4 mA h g^−1^ even at 5 A g^−1^ after 500 cycles. The study reveals a new approach for the design and fabrication of Si-based anodes for high-performance LIBs applications, which will be helpful to the exploitation of various high-performance anodes and may drive the technical development of the combined utilization of dealloying as well as ball milling.

## 2. Results and Discussions

### 2.1. Characterization of Si/Fe_2_O_3_/rGO

**Figure 1:**
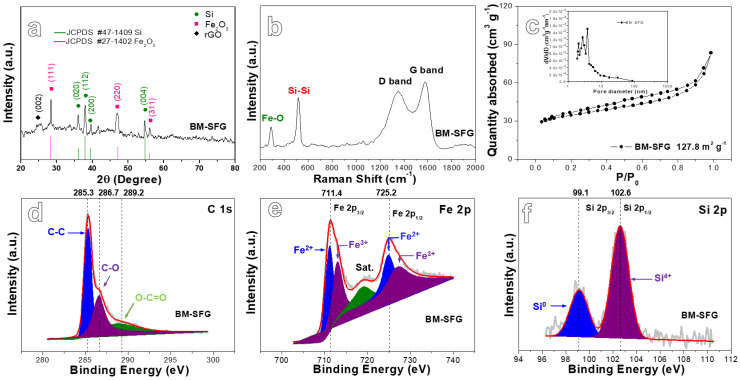
(**a**) X-ray diffraction (XRD) pattern of the BM-SFG; (**b**) Raman spectrum of the BM-SFG; (**c**) N_2_ adsorption-desorption isotherm characteristics and pore size distribution (inset) and high-resolution XPS spectra of (**d**) C 1s, (**e**) Fe 2p, and (**f**) Si 2p for the BM-SFG.

The XRD patterns of the BM48-D4, BM-SFG, and UD-SFG samples are shown in Figure 1a and Figure S1a. The three materials present similar peak positions but are different in peak width and peak intensity. The diffraction peaks located at 28.4°, 47.3°, and 56.1° are related to the (111), (220), and (311) lattice planes of Si (JCPDS No.27-1402) [36,37], while the diffraction peaks at 36.2°, 38.1°, 39.5°, and 54.9° correspond to the (020), (112), (200), and (004) lattice planes of Fe_2_O_3_ (JCPDS No.47-1409) [38,39]. In addition, a weak peak around 25° can be observed in the XRD patterns of BM-SFG and UG-SFG, in accordance with the (002) lattice planes of rGO [40]. It should be emphasized that the first-step ball-milling process changes the element distribution of the dealloying precursor so that the dealloyed product no longer contains Al_9_FeSi_3_ [33] and other intermetallic phases, opening a new door for the design of novel LIB anode materials by tuning the microstructure of dealloying precursors through ball milling.

Figure 1b reveals the Raman spectroscopy of the BM-SFG material. Four peaks can be clearly seen. The peak observed around 520 cm^−1^ corresponds to the Si-Si bond [28,41], while the peak at 293 cm^−1^ is related to the Fe-O bond of Fe_2_O_3_ [42,43]. Two strong peaks at 1200–1600 cm^−1^ are in accord with the D band (defects and disorder) and G band (demonstrating the presence of SP^2−^ hybridized carbon) of rGO [44]. The ratio of peak intensity I_D_/I_G_ is 0.86. Figure S1b reveals Raman spectra of rGO, BM48-D4, and UD-SFG. Obviously, the Raman spectrum of rGO only presents the D band and G band of rGO, with an I_D_/I_G_ ratio of 0.82. The Raman spectrum of BM48-D4 presents the Si-Si bond and the Fe-O bond peaks. The Raman spectrum of UD-SFG shows the D band and G band of rGO with an I_D_/I_G_ ratio of 0.84 as well as the Si-Si bond and the Fe-O bond peaks. In this situation, the peak intensities of the Si-Si bond and the Fe-O bond peaks of BM-SFG are slightly stronger than those of UD-SFG, and the I_D_/I_G_ ratio of BM-SFG is higher than that of UD-SFG and rGO, indicating that BM-SFG has a greater degree of defects in all the test samples, favoring ion and electron transmission during cycling. The XRD and Raman results confirm the successful preparation of the Si/Fe_2_O_3_/rGO composites.

The nitrogen adsorption–desorption isotherm of BM-SFG is displayed in Figure 1c. It shows a typical type-III isotherm with a type-H3 hysteresis loop [45], revealing the presence of mesopores. The BM48-D4 and UD-SFG samples show a similar curve type, with BM-SFG in isotherms (Figure S2a,b). The specific surface area of BM-SFG is 127.8 m^2^g^−1^, which is much higher than for the BM48-D4 (38.4 m^2^g^−1^) and UD-SFG (45.8 m^2^g^−1^) samples. The pore size distribution of the three samples (Figure 1c and Figure S2c) are concentrated at 2–5 nm, further revealing the plentiful mesopores in the test materials. The existence of these mesopores can adapt to the volume variation of materials in the cycling process and improve the transmission speed of Li ions by shortening the diffusion path [46]. With a higher specific surface area, BM-SFG is expected to show better electrochemical performance.

The superficial elements and valence states of the materials were revealed by XPS. The full XPS spectrum presented in Figure S3a uncovers the existence of Si, Fe, C, Al, and O elements in the composites. The O 1s spectrum (Figure S3b) can be divided into two peaks. The peak at 532.8 eV is from hydroxyl, while the peak at 531.8 eV can be attributed to the peak of metal bond in oxide, namely the Fe-O bond (OM bond) in Fe_2_O_3_ [47]. Figure 1d and Figure S3c display the C 1s spectra of BM-SFG and UD-SFG, respectively. The peaks at 285.3, 286.7, and 289.2 eV are in accord with C-C, C-O, and O-C=O, respectively [48,49]. Figure 1e and Figure S3d present Fe 2p maps, showing three characteristic peaks concentrated at 711.4 eV, 725.2 eV, and 719.3 eV, relating to the Fe 2p_3/2_, Fe 2p_1/2_, and satellite peaks, respectively. The energy difference for Fe 2p_1/2_ and Fe 2p_3/2_ is of 13.8 eV, demonstrating the formation of Fe_2_O_3_ [50,51]. The Si 2p spectra of Figure 1f and Figure S3e reveal two characteristic peaks of Si, which are located at 99.1 and 102.6 eV, corresponding to Si^0^ and Si^4+^, respectively [52], showing a slight oxidation of superficial Si. Al peak can be observed in this situation (Figure S3f), indicating that there was still uncorroded Al in the materials after dealloying. Based on the above results, we can conclude that the final product consists of Si, Fe_2_O_3_, rGO, and residual Al. The mass ratio of Si/Fe_2_O_3_/rGO composites was estimated by considering XPS, EDS, and ICP-MS results. It was found that the mass ratio of Si:Fe_2_O_3_:rGO:Al was close to 68.9:15.8:13.6:2.7.

**Figure 2:**
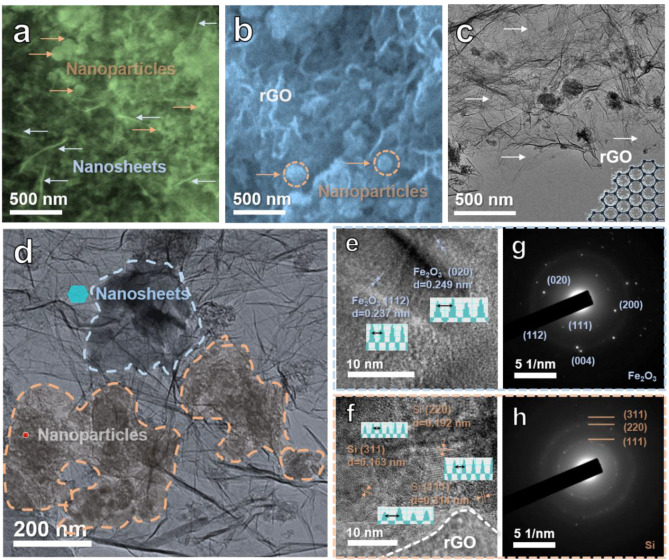
SEM images of (**a**) BM48-D4 and (**b**) BM-SFG. (**c**–**f**) TEM images of BM-SFG at different magnifications. (**g**,**h**) The corresponding selected-area electron diffraction patterns.

SEM images of the BM48-D4 and BM-SFG samples are shown in Figure 2a,b, respectively. Two main morphologies, nanoparticles and nanosheets, can be easily detected (Figure 2a and Figure S4a). Based on previous results [35], the nanosheets relate to Fe_2_O_3_, while the nanoparticles correspond to Si. It was found that BM48-D4 contains Fe_2_O_3_ nanosheets and Si nanoparticles. Although the two materials are uniformly distributed, they lack a network with good electrical conductivity. After ball milling, the Fe_2_O_3_ nanosheets and Si nanoparticles are covered with rGO network (Figure 2b). Figure S4b displays the SEM image of the UD-SFG composite, showing clear coarsening of Fe_2_O_3_ nanosheets and rGO frameworks after the ultrasonic process.

**Figure 3:**
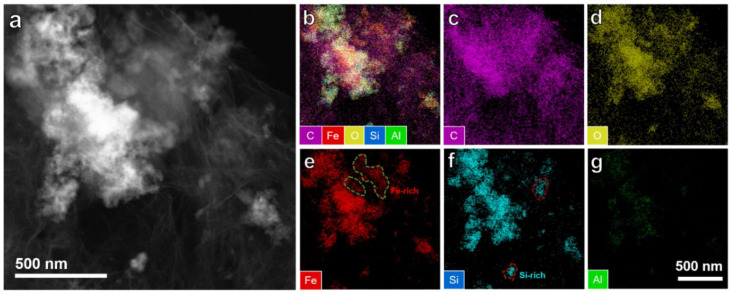
(**a**) STEM image. (**b**–**g**) Elemental mapping of BM-SFG: (**c**) C, (**d**) O, (**e**) Fe, (**f**) Si, (**g**) Al.

TEM images (Figure 2c,d) show that the Fe_2_O_3_ nanosheets and Si nanoparticles are uniformly anchored on rGO networks. In this situation, the three materials contact each other in pairs, ensuring the continuity of the conductive channel and the high conductivity of the composites conducive to improving the electrochemical performance of LIBs. The HRTEM images reveal the lattice fringe of the nanosheet region and the nanoparticle region (Figure 2e,f, respectively). The lattice fringe in Figure 2e shows crystal plane spacing of about 0.249 nm and 0.237 nm, in accordance with the (020) and (112) planes of Fe_2_O_3_ [53]. The lattice fringe in Figure 2f presents interplanar spacing of 0.314 nm, 0.192 nm, and 0.163 nm, relating to the (111), (220), and (311) planes of Si [54]. The selected area electron diffraction patterns (Figure 2g,h) toward the nanosheet region and the nanoparticle region are also in accord with the characteristics of Fe_2_O_3_ and Si, respectively. TEM and corresponding elemental mapping images of the BM-SFG sample are presented in Figure 3. The C element is permeated throughout the sample, relating to rGO networks, which ensure an improved conductivity. In addition, the Fe-rich and Si-rich areas can be found in Figure 3e,f, respectively, corresponding to Fe_2_O_3_ and Si, respectively. All the tests confirm the successful preparation of Fe_2_O_3_ nanosheets and the Si nanoparticle-anchored rGO network.

### 2.2. Electrochemical Performance of Li-Ion Batteries

**Figure 4:**
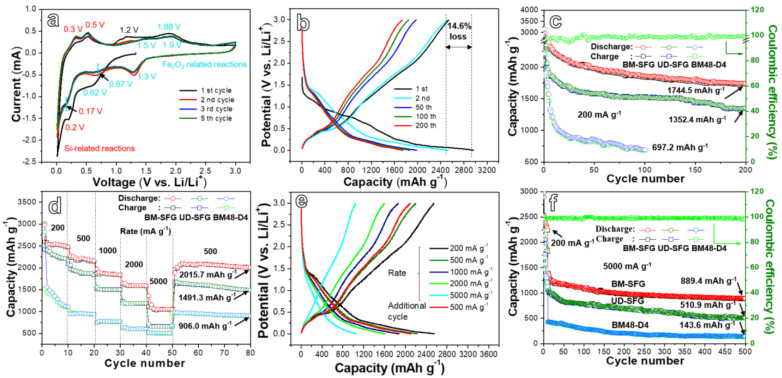
(**a**) CV curves of the BM-SFG electrode measured at 0.1 mV s^−1^ between 0.01 and 3 V. (**b**) Galvanostatic charge-discharge (GCD) profiles of the BM-SFG electrode recorded under 200 mA g^−1^. (**c**) Cyclic performances of the BM-SFG, UD-SFG, BM48-D4 anodes at a current density of 200 mA g^−1^. (**d**) The rate performance of the BM-SFG, UD-SFG, and BM48-D4 electrodes. (**e**) The charge/discharge profiles of BM-SFG, UD-SFG, and BM48-D4 electrodes recorded at different scan rates. (**f**) Cycling performance at 5000 mA g^−1^.

The CV curve measurement of the BM-SFG composite was carried out in the range of 0.01–3.0 V at 0.1 mV s^−1^, as shown in Figure 4a. In the first reduction process, a strong reduction peak at 0.62 V is observed, relating to the conversion of Fe^3+^ to Fe^0^ (Fe_2_O_3_ + 6Li^+^ + 6e^−^ → 3Li_2_O + 2Fe) [55] and the formation of the solid electrolyte interface (SEI) film [56]. Obvious reduction peaks at 0.2 V and a steep peak in the range of 0.01~0.15 V are attributed to the generation of amorphous Li_x_Si phase deriving from lithiation of crystalline Si. In the anodic sweep, two peaks at 0.3 V and 0.5 V relate to the delithiation of Li_x_Si to form Si. The peak emerging around 1.2 V may arise from the reaction between Li ions and the superficial oxygenic functional groups [57]. A broad peak centered at 1.88 V corresponds to the multi-step oxidation of Fe0 to Fe^2+^ and Fe^2+^ to Fe^3+^ [58]. In the second cathodic scan, a new peak at 1.3 V is formed. In this situation, the reaction from Fe^3+^ to Fe^0^ in the subsequent process may be completed through two routes. In the first possible route, the lithiation of Fe_2_O_3_ (Fe_2_O_3_ + xLi^+^ + xe^−^ → Li_x_Fe_2_O_3_) occurs at the peak of 1.3 V [59]. The reduction of Fe^3+^/Fe^2+^ to Fe^0^ is carried out at the peak of 0.67 V (shifted from 0.62 V in the first scan). In the second possible route, the reduction of Fe^3+^ to Fe^0^ occurs through two steps, in which the reduction of Fe^3+^ to Fe^2+^ takes place at the peak of 1.3 V and the reduction of Fe^2+^ to Fe^0^ occurs at the peak of 0.67 V. More detailed testing is needed in the future to uncover this process. In the second anodic scan, the increase in peak intensity of the two peaks at 0.3 and 0.5 V is derived from the activation of Si. The broad peak around 1.88 V is decomposed into two peaks at about 1.5 V and 1.9 V, corresponding to the two-step oxidation from Fe^0^ to Fe^2+^ and from Fe^2+^ to Fe^3+^, respectively. With the increase in cycle number, the reduction and oxidation peaks shift slightly, which may be caused by the irreversible structural rearrangement of active materials in the lithiation/delithiation process. Moreover, the CV curves display good overlap, indicating a good reversibility of the BM-SFG anode.

Figure 4b and Figure S5 present the galvanostatic charge–discharge curves of the BM-SFG and UD-SFG composites, respectively. The initial discharge curve contains a non-repeatable platform at about 1.2 V, which can be attributed to the decomposition of the electrolyte and the formation of the SEI layer, leading to the irreversible capacity loss of the first cycle. The platform in the range of 0.8~0.6 V relates to the conversion of Fe^3+^ to Fe^0^. Due to the lithiation/Li-insertion process of Si and rGO, an obvious and long inclined platform appears when the discharge curve reaches 0.2~0.01 V [60]. The delithiation reaction of silicon occurs at 0.2~0.6 V in the charging voltage platform. Another long charging platform within the scope of 1.2~2.0 V corresponds to the multi-step oxidation of Fe^0^ to Fe^2+^ and Fe^2+^ to Fe^3+^. These potential platforms are consistent with the potentials toward the chemical reaction peaks in the CV curve. The capacity loss values during the first discharge/charge process of BM-SFG and UD-SFG were 14.6% and 28.6%, respectively. There is no obvious difference in curve shapes among the subsequent cycles, which reflects the good stability of the anode material. With the increase in cycle numbers, the charge–discharge curves of the electrode gradually shift to the left, indicating that the electrode capacity declines during the cycle. After 50 cycles, the charge–discharge curves present good overlap, which indicates that the material possesses good cycling stability in the later cycle.

According to the mass ratio of the active product, the theoretical mass specific capacity of the electrode material (3157.5 mAh g^−1^) can be estimated from Equation (1).

(1) $$C_{\mathrm{theoretical}\;}{=C}_{\mathrm{Si}}\;\frac{68.9}{68.9+15.8+13.6}{+C}_{{\mathrm{Fe}}_{2}O_{3}}\frac{15.8}{68.9+15.8+13.6}{+C}_{\mathrm{rGO}\;}\frac{13.6}{68.9+15.8+13.6}\;$$

where *C*_theoretical_ represents the theoretical capacity of the composite, and C_Si_, C_Fe2O3_, and C_rGO_ represent the theoretical capacity of Si, Fe_2_O_3_, and rGO, respectively. Figure 4c shows the cycling performance of BM-SFG, UD-SFG, and BM48-D4 cycling at 200 mA g^−1^. The specific capacity of these samples decreases rapidly in the first 10 cycles and declines at a slower rate in the subsequent cycles. The first discharge/charge capacities of the BM-SFG, UD-SFG, and BM48-D4 electrodes are 2967.5/2534.8, 2893.4/2093.5, and 3167.5/2234.8 mAh g^−1^, respectively. The initial coulomb efficiency of BM-SFG electrode (85.4%) is higher than that of the UD-SFG (71.4%) and BM48-D4 (70.6%) electrodes. The formation of SEI film leads to the irreversible loss of capacity and consumes part of Li^+^ to form inert lithium. After 200 cycles, the reversible capacities of the BM-SFG, UD-SFG, and BM48-D4 electrodes are 1744.5 mAhg^−1^, 1352.4 mAhg^−1^, and 697.2 mAhg^−1^ (100th cycle), respectively. It can be clearly seen that the BM-SFG electrode shows the highest coulomb efficiency, the best capacity retention, and cycle stability, demonstrating the best comprehensive performance among the tested materials. According to the mass ratio of the active materials, active Si, Fe_2_O_3_, and rGO contribute 70.1%, 16.1%, and 13.8%, respectively, towards the battery performance of the BM-SFG.

The rate performances of the three anodes were measured at different current densities, as shown in Figure 4d. The BM-SFG composite delivers reversible capacities of 2546.7, 2222.9, 1868.9, 1598.6, and 1056.8 mAh g^−1^ at 0.2, 0.5, 1, 2, and 5 A g^−1^, respectively, which is clearly higher than those of UD-SFG and BM48-D4 electrodes. In addition, when the current density recovers to 500 mA g^−1^, the BM-SFG presents a reversible capacity of 2015.7 mAh g^−1^ after cycling for an additional 30 cycles. Figure 4e shows the representative galvanostatic charge/discharge curves of BM-SFG at current densities from 200 to 5000 mA g^−1^. With the increase in current density, the shape of the curve basically remains unchanged, while the position gradually moves to the left. When the current density recovers, two group of galvanostatic charge/discharge curves (500 mA g^−1^) nearly overlap with each other, demonstrating its good recoverability. The long-cycle performances of BM-SFG, UD-SFG, and BM48-D4 at a high current density of 5000 mAh g^−1^ are explored in Figure 4f, presenting discharge capacities of 889.4, 510.9, and 143.6 mAh g^−1^, respectively, after 500 cycles. In this situation, the BM-SFG electrode uncovers the best cycling stability in high current density, demonstrating the structural superiority of as-obtained Si/Fe_2_O_3_-anchored rGO framework.

**Figure 5:**
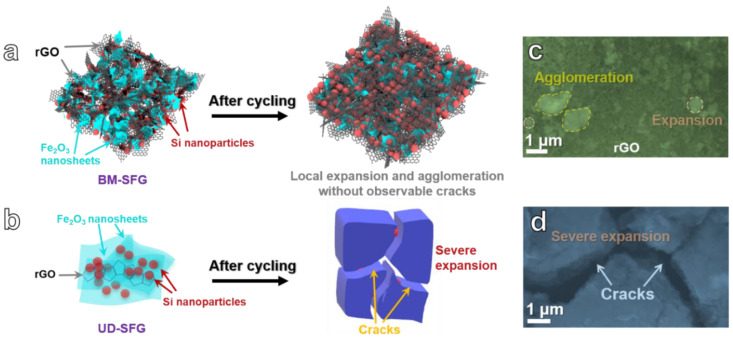
(**a**,**b**) Schematic illustration of the structural evolution before and after cycling: (**a**) BM-SFG, (**b**) UD-SFG. SEM images of the experimental anodes after cycling for 200 cycles at 200 mAg^−1^: (**c**) BM-SFG, (**d**) UD-SFG.

The schematic diagrams reflecting the structural changes of BM-SFG and UD-SFG before and after the cycle are presented in Figure 5a,b, respectively. Moreover, SEM images of two electrodes after cycling for 200 cycles at 200 mA g^−1^ are presented in Figure 5c and d. BM-SFG basically maintains its original morphology after cycling. At the same time, Si nanoparticles and Fe_2_O_3_ nanosheets coarsen, expand, and agglomerate to a certain extent without observable cracks or fractures. However, serious expansion and fracture can be observed in cycled UD-SFG. The great morphological difference of the two electrodes after cycling is due to the large differences in the three-dimensional structure, size, and specific surface area of the initial composites. With higher specific surface area and thinner nanosheets, the three-dimensional BM-SFG framework can well accommodate the volume expansion of active materials, while the coarsened UD-SFG network cannot.

The Raman and XRD results of the BM-SFG electrodes before and after cycling at high current density (5000 mA g^−1^) for 50 cycles are provided in Figure S6. No obvious changes in peak position and intensities of BM-SFG electrodes can be found, proving that the structure and composition of the BM-SFG electrode are relatively stable after cycling. These results further demonstrate the successful synthesis of the Si/Fe_2_O_3_/rGO composite and that the initial material is the material reacting in the cycling.

**Figure 6:**
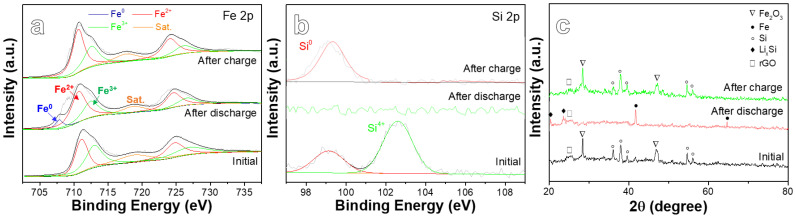
Ex situ XPS spectra analysis of the BM-SFG electrodes at different stages: (**a**) Fe 2p and (**b**) Si 2p. (**c**) Ex situ XRD results of the BM-SFG electrodes at different stages.

Figure 6a,b present an ex-situ XPS spectra analysis of the BM-SFG electrodes at different stages. As shown in Figure 6a, the Fe 2p spectra change significantly after the first discharge. A new peak appears at about 707.9 eV, which is related to the formation of Fe^0^. This fully shows that Fe_2_O_3_ participates in the lithium storage reaction in the charging/discharging process. After the charge, the XPS peak position is restored to the initial position of the material, indicating that the electrode has undergone an effective reversible reaction. As shown in Figure 6b, after the first discharge the peak corresponding to Si 2p becomes weak, which can be attributed to the formation of multi-component mixtures composed of different Li_x_Si products that weaken the XPS signal of a single component, indicating that the alloying reaction between Si and Li took place during the cycling. After charging, only the peak of Si^0^ can be found, corresponding to the delithiation reaction of Li_x_Si to Si. Figure 6c presents ex situ XRD results of the BM-SFG electrodes at different stages. After the first discharge, the main peak intensity of Si is markedly lower than that of the original material. Li_x_Si peaks appear at 20.2° and 23.5°, while Fe peaks appear at about 42.5° and 65°, in accordance with the lithiation of Si to Li_x_Si and the reduction of Fe^3+^ to Fe, respectively. After charging, the XRD peak position is returned to the initial position of the material, demonstrating a good reversibility of the materials.

**Figure 7:**
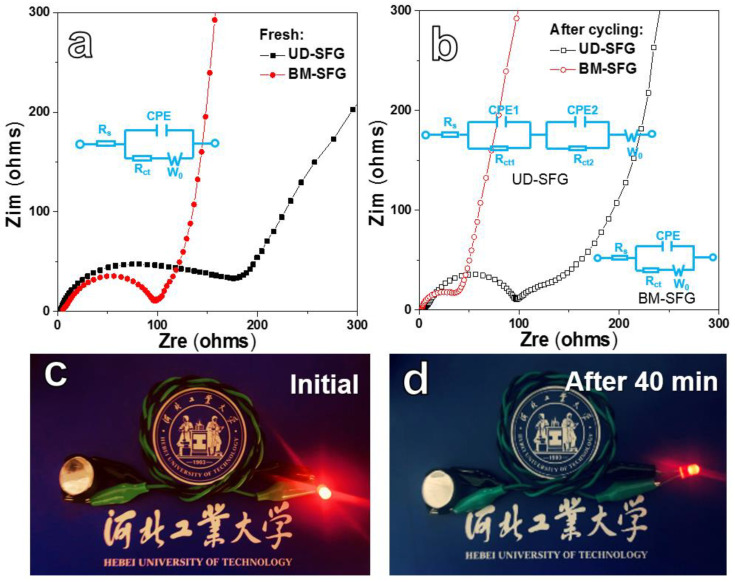
Nyquist plots for BM-SFG and UD-SFG electrodes: Fresh (**a**) and after 100 cycles (**b**). Digital photographs of a red LED bulb propelled by the BM-SFG battery: Initial (**c**) and after 40 min (**d**).

The EIS spectra of BM-SFG and UD-SFG electrodes before and after cycling for 200 cycles are exhibited in Figure 7a,b. The Nyquist plots consist of two parts: a semicircle in the high- and medium-frequency area corresponding to charge transfer resistance and a slash in the low-frequency area caused by ion diffusion. Obviously, the BM-SFG electrode presents lower charge transfer resistance than the UD-SFG electrode, which may benefit from the well-weaved rGO frameworks. The charge transfer resistance of two electrodes decreases notably after cycling, which may be caused by the structural rearrangement of active materials and the generation of stable SEI layer [61,62,63] during cycling. In addition, the BM-SFG electrode still presents a lower charge transfer resistance than the UD-SFG electrode after cycling. The detailed fitted data of EIS are provided in Table S1. These results reveal the structural advancement of the BM-SFG framework, which may induce a lower resistance and better conductivity. A digital photo of a red light-emitting diode (LED) powered by a half cell is shown in Figure 7c. After 40 min, the LED bulb reduces its brightness (Figure 7d) but still works, showing the potential application of the BM-SFG electrode in the energy storage field.

### 2.3. Comparison of Electrochemical Performance of Li-ion Batteries

Table 1 displays the electrochemical performance of different Si-based electrode materials [26,28,64,65,66,67,68,69,70]. The as-synthesized BM-SFG anode presents relatively excellent Li storage performance, which is better than that of many reported Si-based composites. The good performance can be ascribed to the following aspects. Firstly, the porous framework can provide a large contact area and interaction between the electrolyte and the active substance, and can further enhance the ionic fluidity and permeability, which is beneficial to the smooth progress of electrochemical reactions. Secondly, the BM-SFG framework with a high specific surface area can buffer volume expansion to avoid the pulverization and separation of the active material from the conductive network and improve the electrochemical stability. Thirdly, the rGO framework provides a fast transmission network for electrons, while the porous structure is helpful for ion transport, which guarantees good rate performances. The paper provides us with a simple method for preparing high performance Si-based anodes. The as-adopted route can be extended to produce more new materials with high Li storage performance.

## 3. Materials and Methods

**Figure 8:**
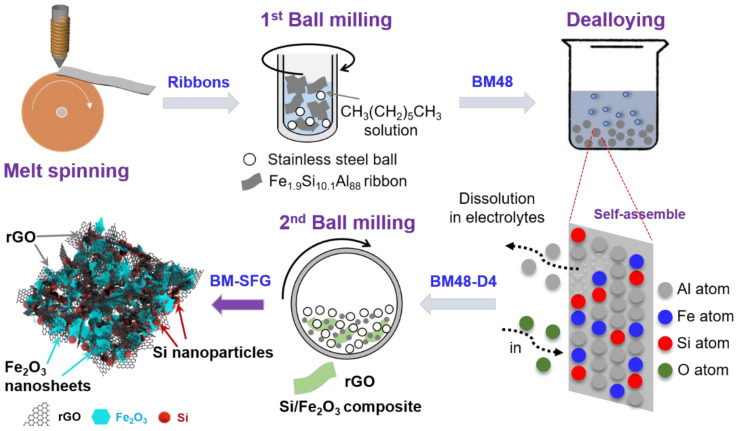
Schematic diagram for the preparation of the BM-SFG.

The typical preparation route of the target product is shown in Figure 8. The Fe_1.9_Si_10.1_Al_88_ ingots were firstly fabricated by our previously reported method [33] through the arc-melting of Al ingots and the ferrosilicon ore (27.15 wt% Fe, 0.49 wt%-associated elements including Mn, S, P, and C, and Si balance) directly. The ingots were re-melted and produced into ribbons by the melt-spinning method [71,72,73] and then were ball-milled (first ball-milling) into powders with a rotation speed of 600 r/min at room temperature for 48 h with ball-to-ribbon ratio of 60:1 (120 g grinding balls, 2.0 g ribbons). To inhibit samples from oxidation during ball-milling, the ribbons were immersed into n-heptane in the tank. The as-obtained powders were washed with absolute ethyl alcohol to remove antioxidants and dealloyed [74,75,76] in 1.25 M NaOH solution for 4 h. After washing in anhydrous ethanol and drying in vacuum oven at 60 °C for 12 h, the dealloyed material (BM48-D4) was synthesized. Then, the BM48-D4 material was mixed with rGO in accordance with the mass ratio of 5:1 and ball milled (second ball-milling) for 10 min under the same milling conditions. After cleaning, the ball-milled Si/Fe_2_O_3_/rGO (BM-SFG) composite was obtained by drying at 60 °C for 12 h under vacuum conditions. For a comparison, the BM48-D4 material and rGO (mass ratio, 5:1) were mixed in the anhydrous ethanol with ultrasonic processing. Thus, the ultrasonic-treated Si/Fe_2_O_3_/rGO (UD-SFG) contrast sample was prepared after vacuum drying. The mass loading of the electrode materials was approximately 1.0~1.2 mg cm^−2^. Experimental details in material characterization and electrochemical measurements can be found in the Supporting Information S0.

## 4. Conclusions

A porous Si/Fe_2_O_3_/rGO composite was successfully prepared using a combined process involving melt-spinning, dealloying, and ball-milling. Si/Fe_2_O_3_ was anchored on the rGO framework to form a three-dimensional porous cross-link structure. Due to this special structure, the electrode revealed a reversible capacity of 1744.5 mAh g^−1^ at 200 mA g^−1^ after 200 cycles and 889.4 mA h g^−1^ at a high current density of 5 A g^−1^ after 500 cycles, presenting excellent cycling stability, high rate properties, and great potential as LIB anode. By involving ball-milling in the dealloying-based preparation process, the composition and structure of the product could be regulated, uncovering a novel strategy for developing high-performance LIB anode materials.

## Figures and Tables

**Table 1:** Comparison of the electrochemical properties of Si-based composites for Li-ion batteries.

Materials	Electrolyte	Current Density (mA g^−1^)	Cycle Number	Reversible Capacity (mAh g^−1^)	Ref.
Si/Ti_3_C_2_ MXene	LX-025 from *DuoDuoChem*	100	200	1475	[64]
Si-Cu_3_Si-CNT/G-C	1 M LiPF_6_ in EC/DEC (1:1) with 10 wt% FEC	200	100	1088	[65]
N-rGO/C@Si	1 M LiPF_6_ in DMC/EC/DEC (1:1:1) with 2 wt% VC and 10 wt% FEC	420	150	1115.8	[28]
Si@Void@C/rGO	1 M LiPF_6_ in EC/DEC (1:1)	500	100	1294	[26]
Si@C@Cu	1 M LiPF_6_ in EC/DMC (1:1) with 2 wt% VC	500	200	1773	[66]
Si/multilayer graphene	1 M LiPF_6_ in EC/DMC/DEC (1:1:1) with 10 wt% FEC	1000	500	990	[67]
Si/Ti_3_C_2_ MXene	LX-025 from *DuoDuoChem*	1000	800	973	[64]
Mg-coated Si film	1.2 M LiPF_6_ in EC/EMC (3:7) with 10 wt% FEC	4200	500	~2100	[68]
Si/TiSi_2_ heteronanostructure	1 M LiPF_6_ in EC/DMC (1:1)	8400	100	937	[69]
Interconnected Si Nanowires	1.15 M LiPF_6_ in EC/DEC (3:7)	8400	70	~1800	[70]
Si/Fe_2_O_3_/rGO (BM-SFG)	1 M LiPF_6_ in EC/DMC (1:1)	200	200	1744.5	This work
		5000	500	889.4	

## Data Availability

The data presented in this study are available on request from the corresponding author.

## Supplementary Materials

The following are available online at https://www.mdpi.com/article/10.3390/ijms222011041/s1.
