# Supplementary material for: Synthesis of Si/Fe2O3-Anchored rGO Frameworks as High-Performance Anodes for Li-Ion Batteries
Source: Int J Mol Sci. 2021 Oct 13;22(20):11041. doi: 10.3390/ijms222011041 (PMC8539548; doi:10.3390/ijms222011041)
Supplement: Supplementary file 1 [file ijms-22-11041-s001.zip › ijms-1382443-SI.pdf]

Supplementary

Synthesis of Si/Fe_2O_3 anchored rGO frameworks as high performance anodes for Li-ion batteries

Yajing Yan [1,2,†], Yanxu Chen [1,†], Yongyan Li [1,*], Xiaoyu Wu [1], Chao Jin [1] and Zhifeng Wang [1,2,3,*]

[1] School of Materials Science and Engineering, Hebei University of Technology, Tianjin 300401, China;
[2] Key Laboratory for New Type of Functional Materials in Hebei Province, Hebei University of Technology, Tianjin 300401, China
[3] Research Institute of Foundry, Hebei University of Technology, Tianjin 300401, China
* Correspondence: liyongyan@hebut.edu.cn (Y.L.); wangzf@hebut.edu.cn (Z.W.)
† These authors contributed equally to this work.

S0: Experimental Section

S01. Material characterization

The phase composition of the products was uncovered by X-ray diffraction (XRD, D8-Discover, Bruker). Scanning electron microscope (SEM, JSM-7100F) and transmission electron microscope (TEM, JEM-2100F) were used to reveal the microstructure of the samples. X-ray photoelectron spectroscopy (XPS, ESCALAB 250XI) was used to analyze the valence of the sample. Nitrogen adsorption-desorption isotherm was performed on Micromeritics Tristar II 3020. The specific surface was analyzed by Brunauer-Emmett-Teller (BET) method, and Barrett-Joyner-Halenda (BJH) method was performed to determine pore size distributions. Raman spectroscopy (inVia Reflex) was measured in the wave number range of 100~2000 cm^{-1}.

S02. Electrochemical measurements

The active materials, carboxymethyl cellulose (CMC) and conductive Super P (mass ratio, 8:1:1) were mixed in deionized water to form a black slurry. Copper foil was coated with slurry and dried at 60°C for 12 h in a vacuum furnace. Then, it was cut into a circular piece with a diameter of 10 mm and used as the working electrode. The electrode was assembled into a CR2032 button battery in a vacuum glove box filled with argon gas. Celgard 2320 was selected as the separator and 1 M $LiPF_6$ dissolved in carbonate (EC)/Diethyl carbonate (DEC) solution (v:v=1:1) was adopted as the electrolyte. The electrochemical impedance spectroscopy (EIS) and cyclic voltammetry (CV) curves of the batteries were conducted using the electrochemical workstation

(CHI760E). Galvanostatic charge-discharge curves were tested on a NEWARE battery tester in the voltage window of 0.01–3 V (vs Li^+/Li).

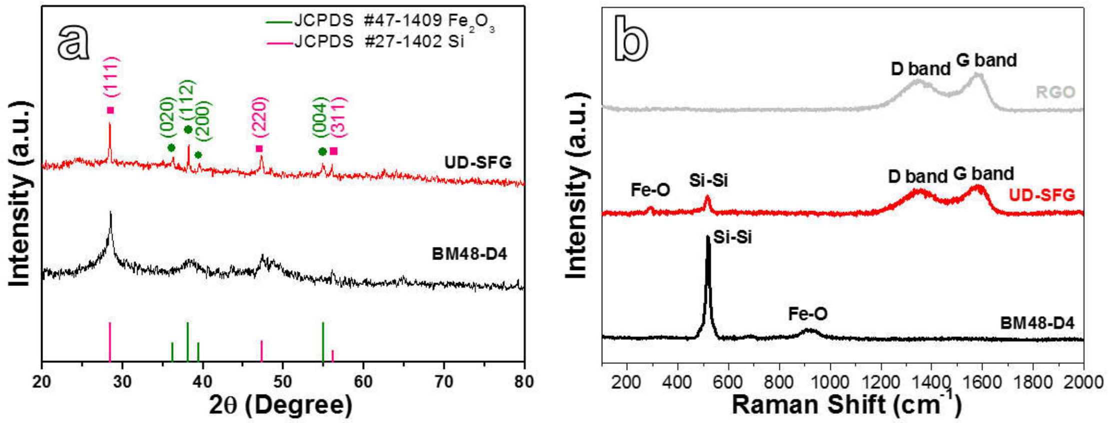

Figure S1 (a) XRD patterns of the UD-SFG and BM-48-4; (b) Raman spectrum of the BM-48-4, UD-SFG and BM-48-4.

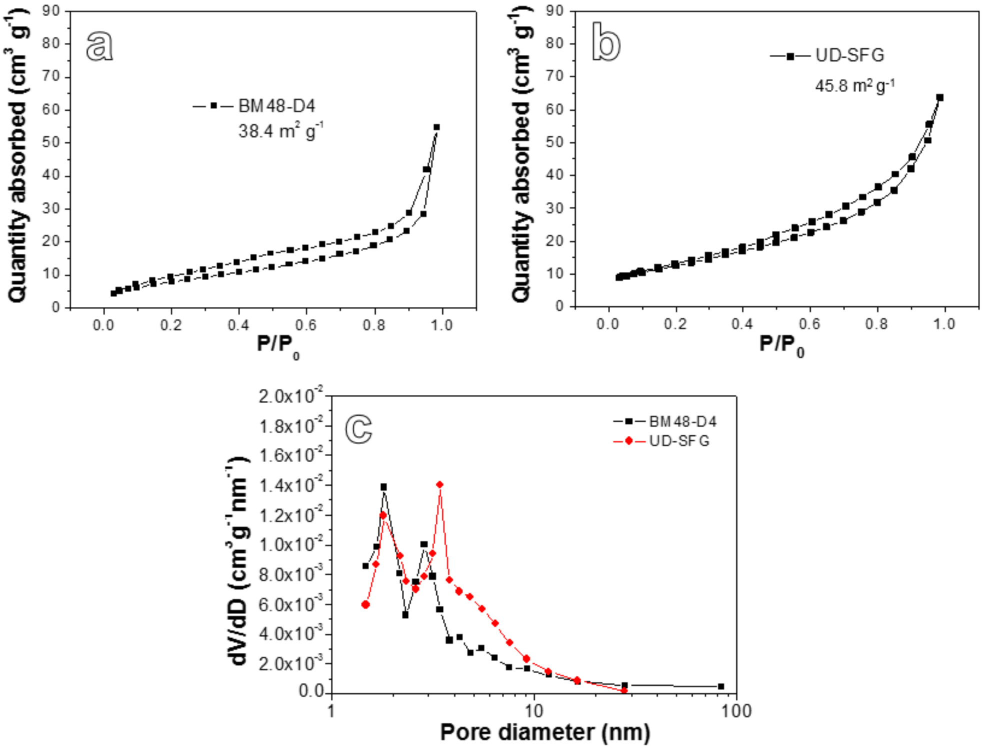

Figure S2 N_2 adsorption-desorption isotherm characteristics of BM48-D4 (a) and UD-SFG (b); (c) Pore size distribution of BM-48-4 and UD-SFG.

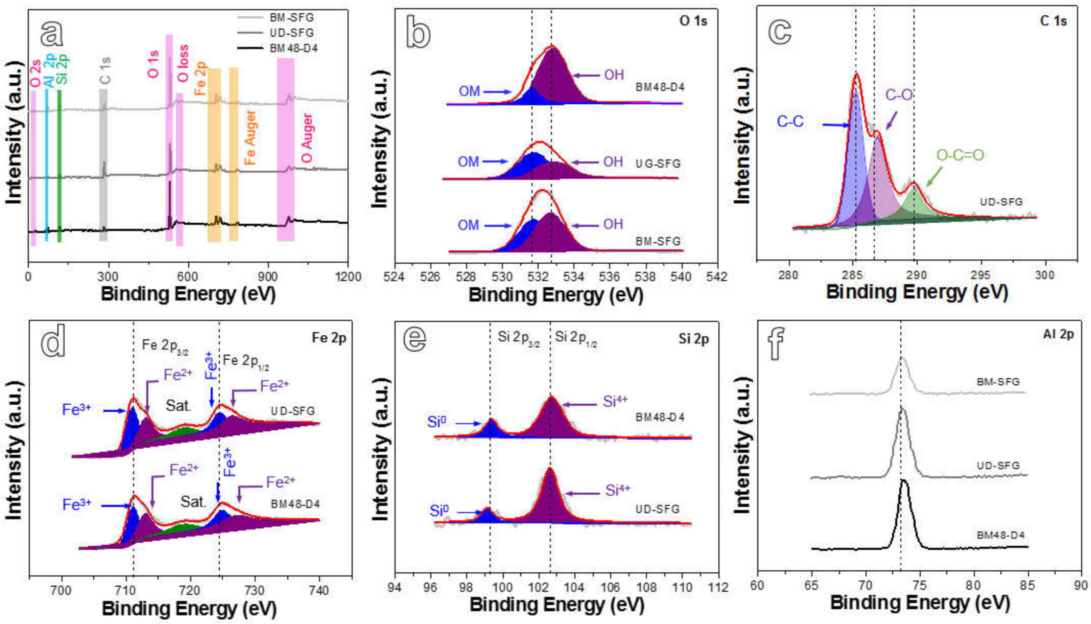

Figure S3 (a) XPS full survey spectrum and XPS spectra of (b) O 1s and (c) Al 2p for the BM-SFG, UD-SFG and BM-48-4; high resolution XPS spectra of (d) C 1s of UD-SFG, (e) Fe 2p and (f) Si 2p for the UD-SFG and BM-48-4.

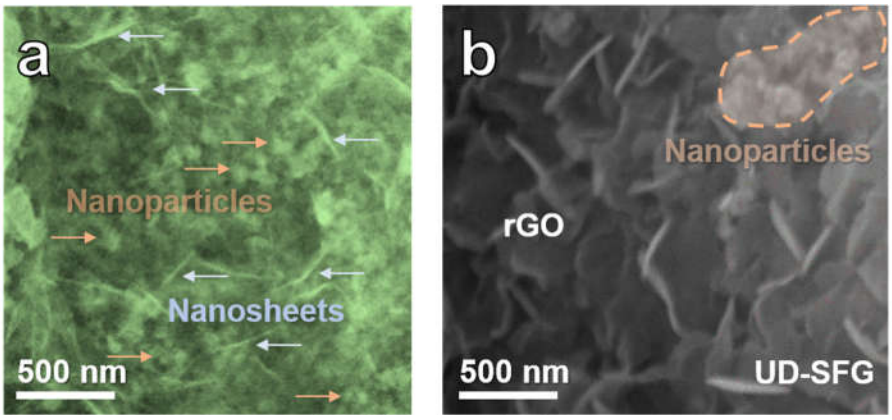

Figure S4 SEM images of (a) BM48-D4 and (b) UD-SFG.

S 3.2

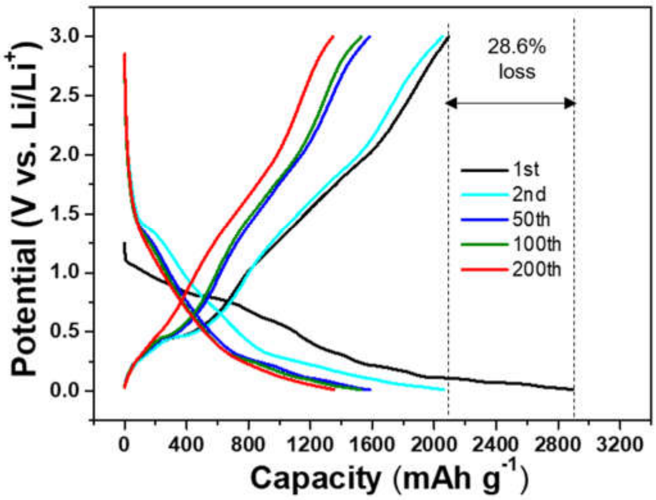

Figure S5 Galvanostatic charge-discharge (GCD) profiles of the UD-SFG electrode recorded under 200 mA g^{-1}.
